# Search and sequence analysis tools services from EMBL-EBI in 2022

**DOI:** 10.1093/nar/gkac240

**Published:** 2022-04-12

**Authors:** Fábio Madeira, Matt Pearce, Adrian R N Tivey, Prasad Basutkar, Joon Lee, Ossama Edbali, Nandana Madhusoodanan, Anton Kolesnikov, Rodrigo Lopez

**Affiliations:** European Molecular Biology Laboratory, European Bioinformatics Institute (EMBL-EBI), Wellcome Trust Genome Campus, Hinxton, Cambridge CB10 1SD, UK; European Molecular Biology Laboratory, European Bioinformatics Institute (EMBL-EBI), Wellcome Trust Genome Campus, Hinxton, Cambridge CB10 1SD, UK; European Molecular Biology Laboratory, European Bioinformatics Institute (EMBL-EBI), Wellcome Trust Genome Campus, Hinxton, Cambridge CB10 1SD, UK; European Molecular Biology Laboratory, European Bioinformatics Institute (EMBL-EBI), Wellcome Trust Genome Campus, Hinxton, Cambridge CB10 1SD, UK; European Molecular Biology Laboratory, European Bioinformatics Institute (EMBL-EBI), Wellcome Trust Genome Campus, Hinxton, Cambridge CB10 1SD, UK; European Molecular Biology Laboratory, European Bioinformatics Institute (EMBL-EBI), Wellcome Trust Genome Campus, Hinxton, Cambridge CB10 1SD, UK; European Molecular Biology Laboratory, European Bioinformatics Institute (EMBL-EBI), Wellcome Trust Genome Campus, Hinxton, Cambridge CB10 1SD, UK; European Molecular Biology Laboratory, European Bioinformatics Institute (EMBL-EBI), Wellcome Trust Genome Campus, Hinxton, Cambridge CB10 1SD, UK; European Molecular Biology Laboratory, European Bioinformatics Institute (EMBL-EBI), Wellcome Trust Genome Campus, Hinxton, Cambridge CB10 1SD, UK

## Abstract

The EMBL-EBI search and sequence analysis tools frameworks provide integrated access to EMBL-EBI’s data resources and core bioinformatics analytical tools. EBI Search (https://www.ebi.ac.uk/ebisearch) provides a full-text search engine across nearly 5 billion entries, while the Job Dispatcher tools framework (https://www.ebi.ac.uk/services) enables the scientific community to perform a diverse range of sequence analysis using popular bioinformatics applications. Both allow users to interact through user-friendly web applications, as well as via RESTful and SOAP-based APIs. Here, we describe recent improvements to these services and updates made to accommodate the increasing data requirements during the COVID-19 pandemic.

## Graphical Abstract

**Graphical Abstract:**
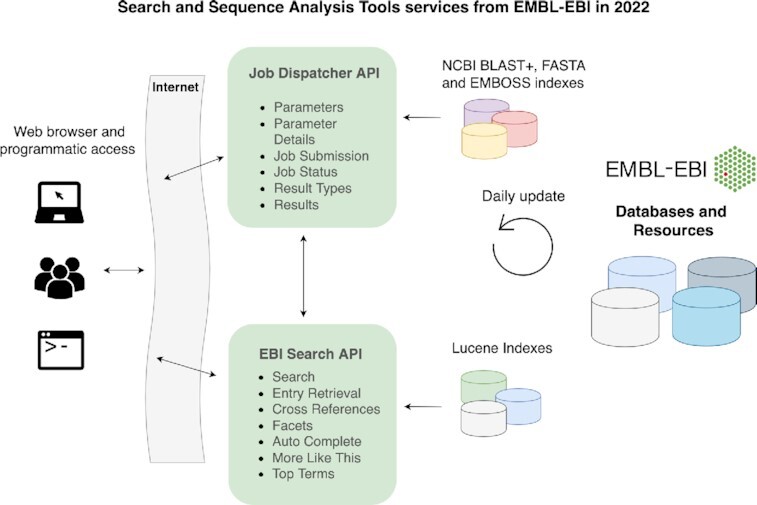
Overview of the tools and data resources provided by EBI Search and Job Dispatcher services accessible via their webpage and programmatic interfaces.

## INTRODUCTION

The COVID-19 pandemic caused by the severe acute respiratory syndrome cCoronavirus 2 (SARS-CoV-2) and the lockdown measures implemented by governments worldwide to contain it has caused unprecedented economic and societal disruption (1). This has highlighted the need for the scientific community to work together to effectively tackle the global COVID-19 health crisis. The European Bioinformatics Institute (EMBL-EBI; https://www.ebi.ac.uk/) alongside many other institutions, have promptly made international cooperation networks to provide researchers and the general public access to trustworthy information. In fact, the pandemic has posed an enormous challenge to biological data resources since 2020, with increasing quantities of data being generated and needing to be made available to users from across the scientific communities (2). EMBL-EBI has contributed to the fight against COVID-19 on several fronts, in particular by helping the development of the European COVID-19 Data Portal (3), which leverages and brings together biomolecular data from a variety of EMBL-EBI’s data resources and services to researchers, clinicians and public health professionals. Additionally, the EMBL-EBI has over the last thirteen plus years developed Web Service API-centred frameworks, EBI Search and Job Dispatcher (4), for providing access to (i) a free text search and powerful cross-referencing engine and to (ii) bioinformatics sequence analysis tools, respectively, that provide access to these rich data. The services have worked closely with teams across the EMBL-EBI and further afield to expand the capabilities of the frameworks. In this paper, we overview recent improvements and updates made to the services to accommodate the increasing data requirements during the COVID-19 pandemic.

## EBI SEARCH AND JOB DISPATCHER

EBI Search (previously EB-eye) is an Apache Lucene-based search engine platform, providing simple and uniform access to the public biological data resources hosted by EMBL-EBI. These data are spread across 160+ data sets (domains), updated daily. Searches may be carried out using a RESTful API or the EBI Search website, returning result sets including hierarchical facets and cross-references to other datasets, allowing links to be followed throughout the available resources. The EBI Search engine provides search functionality to services across EMBL-EBI, including ENA (5), Ensembl Genomes (6), the OMICS DI portal (7) and the COVID-19 Data Portal.

The Job Dispatcher tools framework (JD) provides integrated access to core bioinformatics applications and required biological data. The JD catalogue of tools includes some of the most popular powerhouses in bioinformatics, from sequence similarity search applications, such as NCBI BLAST+ (8) and FASTA (9), multiple sequence alignment and pairwise sequence alignment tools, such as Clustal Omega (10) and Kalign (11), tools for functional annotation and prediction such as InterProScan 5 (12), RNA analysis tools such as R2DT (13), to other sequence analysis utilities. The use of sequence similarity search tools comprises 45 000+ distinct sequence libraries from major database resources hosted at EMBL-EBI, including UniProtKB (14), ENA and Ensembl Genomes. The JD framework provides an interface between high-performance compute clusters and command-line applications. Free access to the tools is provided via the service's website, as well as programmatically, via transparent and reliable RESTful and SOAP Web Services APIs. Visual representations of tool results are also provided to help the users understand the job outputs. An additional component of the JD offering is Dbfetch, which provides a common interface to database entry retrieval in a variety of different formats for all the sequence libraries available to search in JD.

## UPDATES ON DATA RESOURCES

EBI Search data resources are grouped into a hierarchical tree of domains (see a list of data resources available in EBI Search in Supplementary Table 1). Since the last update, Open Targets (15), VarSite (16), PDBe-KB (17), GWAS Catalog (18), EMPIAR (19), European Variation Archive Studies (20) and Cellosaurus (21) have been added as new resources. Additionally, 18 new COVID-19-specific domains have been added.

New sequence libraries were added to JD (see a list of all the sequence libraries currently provided by JD in Supplementary Table 2), namely SARS-CoV-2 dataset releases from Ensembl, UniProtKB, ENA and Pfam (22). IPD-NHKIR (23) coding and genomic sequences, as well as sequences from the AlphaFold DB (24), EMDB (25) and PDBe-KB, have also been made available for sequence similarity search in the JD tools framework and retrieval via Dbfetch.

## UPDATES ON THE TOOLS

Sequence analysis tools running under JD are categorised according to their functionality and have been regularly updated to their latest available versions (see a list of all the categories and bioinformatics tools currently provided by JD in Supplementary Table 3). These were also updated to run in containers with Singularity that future-proof their execution and isolation in an ever-changing computational environment. Three new tools have been added to the framework since the last update, including R2DT, for predicting and visualising RNA secondary structures, SSEARCH2SEQ and GGSEARCH2SEQ, for generating local and global pairwise alignments, respectively (9). A new JSON schema (https://github.com/ebi-wp/sss_json_schema) has been developed enabling a standardised JSON output to be provided for the results of sequence searches by tools in the FASTA and NCBI BLAST+ suites.

## USAGE OF THE SERVICES DURING THE COVID-19 PANDEMIC

The EBI Search API has been providing the search results for the COVID-19 Data Portal since that site's inception in April 2020, with the team closely involved with the site's development. Data is retrieved from a combination of domains dedicated to purely COVID-19-related data and wider domains queried with filters applied to limit content. Changes have been made to the indexing process to allow data to be copied between domains, de-normalizing data and improving response times. Deep paging (https://lucidworks.com/post/coming-soon-to-solr-efficient-cursor-based-iteration-of-large-result-sets/) has been enabled in selected COVID-19 domains, enabling search results to be retrieved beyond the one million result limit to assist with data verification. A base web application has been developed from the COVID-19 Data Portal for future EMBL-EBI portal projects specialising in particular areas of health bioinformatics that cut across multiple datasets.

The deployment model and reliability of JD data pipelines for daily indexing of biological databases has been improved. These are now generated using BLAST database version 5 which together with NCBI Taxonomy data (26), enables limiting the search by taxonomy with both inclusion and exclusion lists of NCBI Taxonomy identifiers (TaxIDs). The NCBI Taxonomy database is updated daily, and a tree structure is built using Taxonomy Resolver (https://github.com/ebi-wp/taxonomy-resolver), based on the NCBI Taxonomy Database classification. This functionality is currently available for sequence searches against UniProtKB and ENA databases.

EBI Search and JD are core services used extensively by other resources and portals at the EMBL-EBI and elsewhere. The sheer volume of data being generated during the COVID-19 pandemic has resulted in an average of 2.5 million requests per day to the EBI Search engine, and nearly 500 million analyses being performed under the JD tools framework in 2021. These are well in line with what was observed during 2020, where a noticeable surge of >130 million sequence analysis performed during the COVID-19 outbreak months of April and May 2020, and highlights the importance of the services described here. Trends in tool usage and citation data of our most recent article describing the framework (4) since 2019 highlight how core bioinformatics applications for sequence searching and alignment are fundamental for life sciences research and development, ranging from structural biology and drug discovery to immunology and epidemiology. Among many other use cases, JD tools have been used for aiding: the identification of the SARS-CoV-2 proteome; aiding the analysis of the SARS-CoV-2 structural and non-structural proteins, including the spike proteins and analysis of their mediated host cell entry; evaluation of new possible drug targets and development of new drugs, vaccine constructs and antibodies; and mutational spectra analysis and detection of new variants (27–31).

## DISCUSSION

At the time of writing, the ongoing pandemic is driving us to improve our services to better serve the scientific community and react quickly to global changes in data demand. The EBI Search team will continue to work on the baseline portal web application to make rapid deployment of future portal sites simple. Improving search performance is a priority, and the team will be applying upgrades to the major software libraries used by the platform. The JD team is currently developing a brand new modern and interactive website and backend for the JD tools framework. Work on an updated tabular results page with new interactive features for sorting, selecting and faceting the results, as well as downloading sequences in bulk and initiating workflows, is underway. We hope these changes will further expand the offering of both tools and datasets while maintaining the security, scalability and reliability of the service.

## DATA AVAILABILITY

EBI Search is available from https://www.ebi.ac.uk/ebisearch and JD tools are available from https://www.ebi.ac.uk/services. Detailed documentation about how to interface with the services programmatically are provided at https://www.ebi.ac.uk/Tools/webservices. Additionally, users can explore the EBI Search and JD APIs interactively at: https://www.ebi.ac.uk/ebisearch/apidoc.ebi and https://www.ebi.ac.uk/Tools/common/tools/help, respectively. Sample Web Service clients in Python, Perl and Java are also provided for EBI Search as well as JD on the following GitHub repositories: https://github.com/ebi-wp/EBISearch-webservice-clients and https://github.com/ebi-wp/webservice-clients, respectively. CWL command-line tool definitions and example workflows are available from https://github.com/ebi-wp/webservice-cwl. These services are developed in accordance with FAIR principles.

## Supplementary Material

gkac240_Supplemental_File
