# Supplementary material for: Search and sequence analysis tools services from EMBL-EBI in 2022
Source: Nucleic Acids Res. 2022 Apr 12;50(W1):W276–9. doi: 10.1093/nar/gkac240 (PMC9252731; doi:10.1093/nar/gkac240)
Supplement: gkac240_Supplemental_File [file gkac240_supplemental_file.pdf]

**Supplementary Table 1** - Data resources available through EBI Search in 2022.

| **Category** | **Data** |
|---|---|
| Genomes and metagenomes | Ensembl Genomes, Ensembl, HGNC, DGVa, EGA, LRG, WormBase ParaSite, MGnify |
| Nucleotide sequences | ENA, RNAcentral, Rfam, NRNL1, NRNL2, IMGT/HLA, IPD-KIR, IPD-NHKIR, IPD-MHC, GWAS Catalog, OMICS ENA Project |
| Protein sequences | UniProtKB, UniParc, UniRef, EPO, JPO, KIPO, USPTO, NRPL1, NRPL2 |
| Macromolecular structures | PDBe, EMDB, EMPIAR, PDBe-KB |
| Bioactive molecules | ChEBI, ChEMBL, Ligands |
| Gene expression | ArrayExpress, Expression Atlases, GEO, dbGaP |
| Molecular interactions | IntAct, Complex Portal |
| Reactions, pathways | Rhea, Reactome, BioModels, MetaboLights, MetabolomeExpress, Metabolomics Workbench, CellCollective, Physiome |
| Protein families | InterPro, TreeFam, Pfam, MEROPS, GPCRDB, HMMER |
| Protein expression data | PRIDE, GNPS, GPMdb, MassIVE, PeptideAtlas, LINCS, Paxdb, jPOST, EVA, Cellosaurus |
| Enzymes | IntEnz, Enzyme Portal |
| Literature | Europe PMC, Patent families |
| Samples and ontologies | Taxonomy, GO, EFO, SBO, MESH, BioSamples, OLS, BioStudies |
| Diseases | OMIM, Human diseases, VarSite |
| Gene-Disease Associations | OpenTargets |
| Catalogs and registries | Bio.tools, FAIRDOMHub, Identifiers.org registry, ORCID data claims |
| Genome variation | EVA Studies |

**Supplementary Table 2** - Sequence libraries available through JD in 2022.

| **Category** | **Data** |
|---|---|
| UniProtKB protein sequences | UniProtKB, SwissProt, SwissProt Isoforms, TrEMBL, UniProtKB Taxonomic Subsets (13 subgroups, including: bacteria, archaea, eukaryota, SARS-CoV-2, etc.), Reference Proteomes, Representative Proteomes (15, 35, 55, 75), UniProt Reference (UniRef 50, 90 and 100), UniParc, Unimes, UniProtKB-PDB |
| Patent protein sequences | EPO, JPO, KIPO, UPSPTO |
| Structures of protein sequences | PDBe, AlphaFold DB |
| Protein families | Pfam, TIGRFAM, Superfamily, Gene3D, PIRSF, TreeFam, Pfam SARS-CoV-2 |
| Other protein sequences | Enzyme Portal, IntAct, IPD-IMGT/HLA, IPD-KIR, IPD-MHC, MEROPS (MP, MPEP and MPRO), ChEMBL, Quest for Orthologs |
| ENA nucleotide sequences | ENA sequences for Coding, Non-coding, Barcode, Geospatial, Ribosomal RNA and others (10 subgroups, including: Expressed Sequence Tag, Genome Survey Sequence, etc.) |
| Ensembl Genomes sequences | Genomes from Bacteria, Fungi, Plants, Metazoa, Protists, WormBase Parasite, SARS-CoV-2 |
| Structures of nucleotide sequences | PDBe |
| Other nucleotide sequences | IMGT/LIGM-DB, IMGT/HLA (CDS and genomic), IPD-KIR (CDS and genomic), IPD-NHKIR (CDS and genomic), IPD-MHC (CDS and genomic) |
| Additional entries available via Dbfetch | EMDB, PDBe-KB, MEDLINE, NCBI Taxonomy, EDAM ontology, HGNC |

**Supplementary Table 3** - Bioinformatics applications available through JD in 2022.

| **Category** | **Tools** |
|---|---|
| Multiple Sequence Alignment (https://www.ebi.ac.uk/Tools/msa/) | Clustal Omega, Kalign, MAFFT, MUSCLE, T-Coffee, MView, WebPrank |
| Pairwise Sequence Alignment (https://www.ebi.ac.uk/Tools/psa/) | Needle, Stretcher, Water, Matcher, LALIGN, GeneWise, GGSEARCH2SEQ, SSEARCH2SEQ |
| Phylogeny Analysis (https://www.ebi.ac.uk/Tools/phylogeny/) | Simple Phylogeny |
| Protein Functional Analysis (https://www.ebi.ac.uk/Tools/pfa/) | InterProScan 5, PfamScan, Phobius, Pratt, RADAR, HMMER3 phmmer, HMMER3 hmmscan |
| RNA Analysis (https://www.ebi.ac.uk/Tools/rna/) | Infernal cmscan, MapMi, R2DT |
| Sequence Similarity Search (https://www.ebi.ac.uk/Tools/sss/) | NCBI BLAST+, PSI-BLAST, FASTA, SSEARCH, FASTM/S/F, GGSEARCH, GLSEARCH, PSI-Search, PSI-Search2 |
| Sequence Statistics (https://www.ebi.ac.uk/Tools/seqstats/) | SAPS, Pepinfo, Pepstats, Pepwindow, Cpgplot, Newcpgreport, Isochore, Dotmatcher, Dottup, Dotpath, Polydot |
| Sequence Translation (https://www.ebi.ac.uk/Tools/st/) | Transeq, Sixpack, Backtranseq, Backtranambig |
| Sequence Format Conversion (https://www.ebi.ac.uk/Tools/sfc/) | Seqret, MView |
| Sequence Operation (https://www.ebi.ac.uk/Tools/so/) | Seqcksum |
| EMBOSS Suite (https://www.ebi.ac.uk/Tools/emboss/) | Needle, Stretcher, Water, Matcher, Transeq, Sixpack, Backtranseq, Backtranambig, Pepinfo, Pepstats, Pepwindow, Cpgplot, Newcpgreport, Isochore, Dotmatcher, Dottup, Dotpath, Polydot, Seqret |
| Database Fetch (https://www.ebi.ac.uk/Tools/dbfetch/) | Dbfetch (fetching data from 57 domains, see Supplementary Table 2) |
